# Treating patients in a trauma room equipped with computed tomography and patients’ mortality: a non-controlled comparison study

**DOI:** 10.1186/s13017-018-0176-3

**Published:** 2018-03-27

**Authors:** Shintaro Furugori, Makoto Kato, Takeru Abe, Masayuki Iwashita, Naoto Morimura

**Affiliations:** 1 Department of Emergency Medicine, Yokohama City University, 4-57 Urafunecho, Minamiku, Yokohama City, Kanagawa Prefecture 232-0024 Japan; 2 Department of Surgery, Yokohama City University, 4-57 Urafunecho, Minamiku, Yokohama City, Kanagawa Prefecture 232-0024 Japan; 3 Department of Acute Medicine, Graduate School of Medicine, The University of Tokyo, 7-3-1 Hongo, Bunkyo-ku, Tokyo, 113-0033 Japan

**Keywords:** Acute care, Trauma resuscitation room, CT

## Abstract

**Background:**

To improve acute trauma care workflow, the number of trauma centers equipped with a computed tomography (CT) machine in the trauma resuscitation room has increased. The effect of the presence of a CT machine in the trauma room on a patient’s outcome is still unclear. This study evaluated the association between a CT machine in the trauma room and a patient’s outcome.

**Methods:**

Our study included all trauma patients admitted to a trauma center in Yokohama, Japan, between April 2014 and March 2016. We compared 140 patients treated using a conventional resuscitation room with 106 patients treated in new trauma rooms equipped with a CT machine.

**Results:**

For the group treated in a trauma room with a CT machine, the Injury Severity Score (13.0 vs. 9.0; *p* = 0.002), CT scans of the head (78.3 vs. 66.4%; *p* = 0.046), CT scans of the body trunk (75.5 vs. 58.6%; *p* = 0.007), intubation in the emergency department (48.1 vs. 30.7%; *p* = 0.008), and multiple trauma patients (47.2 vs. 30.0%; *p* = 0.008) were significantly higher and Trauma and Injury Severity Score probability of survival (96.75 vs. 97.80; *p* = 0.009) was significantly lower than the group treated in a conventional resuscitation room. In multivariate analysis and propensity score matched analysis, being treated in a trauma room with a CT machine was an independent predictor for fewer hospital deaths (odds ratio 0.002; 95% CI 0.00–0.75; *p* = 0.04, and 0.07; 0.00–0.98, respectively).

**Conclusions:**

Equipping a trauma room with a CT machine reduced the time in decision-making for treating a trauma patient and subsequently lowered the mortality of trauma patients.

## Background

Trauma is the leading cause of death among young people around the world [1] and in those aged < 45 years in Japan [2]. In addition, approximately 23,000 trauma deaths occur each year in Japan [2]. Trauma has a negative impact on the lives of people and is a risk for social welfare [1]. Improving therapeutic procedures and diagnostic evaluations for trauma patients is necessary to increase their survival and improve public health.

In recent years, computed tomography (CT) has provided faster operations and more detailed images and can be made easily available in acute trauma care. CT scanning in the early diagnostic phase of trauma care is critical and has become an essential part of a trauma diagnostic work-up. In previous studies, CT scanning contributed to a change of treatment without obvious external signs of injuries [3–5], gained time benefits compared with a conventional resuscitation [6–9], and had potential survival benefits for trauma patients, especially when total-body CT scanning (TBCT) was performed [10–15]. To improve acute trauma care workflow, the number of trauma centers equipped with a CT machine in the trauma resuscitation room has increased [6, 7, 16–20]. Equipping a CT machine in a trauma room is expensive, and the effect of a CT machine in the trauma room compared with a conventional resuscitation room on a patient’s outcome is still unclear because of the inconsistency in previous findings [6, 18–22]. Thus, we conducted a before and after comparison study to evaluate the association between the presence of a CT machine in the trauma resuscitation room and a patient’s outcome.

## Methods

**Fig. 1:**
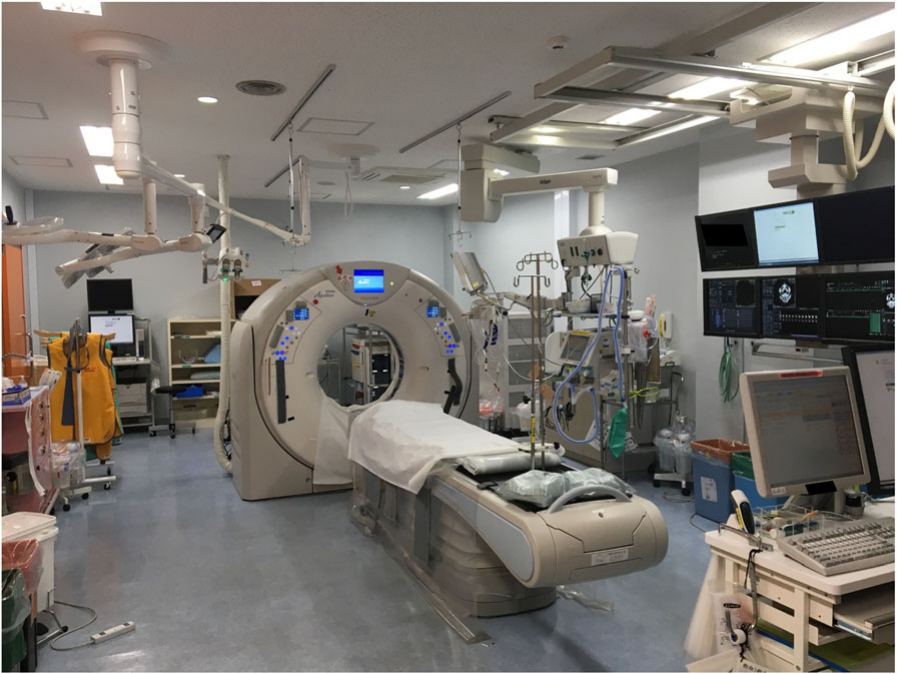
The new trauma resuscitation room at the Yokohama City University Medical Center

The Yokohama City University Medical Center (YCUMC), Yokohama, Japan, has recently equipped a trauma room with a CT machine and has been functioning as a designated, regional trauma center since April 2015. Yokohama City has approximately 3.7 million inhabitants. In April 2015, a prehospital-to-hospital care protocol was introduced. In the protocol, emergency medical services were used to transport severe trauma patients, such as those suffering from shock from a blunt trauma, penetrating injuries to the neck, or two or more proximal long-bone fractures, to a designated trauma center. Regarding pre-hospital care system in Japan, a regional medical control council (MC council) determines treatment and delivery protocols, depending on a patient’s conditions. In this system, a local ambulance transfers severe trauma patients to a tertiary medical facility, and an emergency and critical care center admits the patient. Yokohama City designated two hospitals as regional severe-trauma centers, based on a criteria indicated by the Japanese Association for The Surgery of Trauma, and YCUMC is one of them. Such designations in Yokohama City are the first attempt by a local administration in the country. At the same time, YCUMC placed a CT machine in a new trauma resuscitation room in the emergency department (ED) (Fig. 1). A trauma team was established, consisting of well-trained staffs to provide patients with a trauma survey and treatment. The trauma team has a leader, who is a trauma surgeon or an emergency physician. After the establishment of the new trauma resuscitation room, a patient is directly transferred to the trauma resuscitation room and onto a CT carbon table. Any life-saving procedures, including airway management, chest tube replacement, or emergency laparotomy, can be performed on the CT table. After the life-saving procedures, each leader decides whether or not to perform a CT scan immediately during the primary survey for patients whose vital signs are within an acceptable range, such as percutaneous oxygen saturation (SaO_2_), 90%; heart rate (HR), 130 bpm; and systolic blood pressure (SBP), 70 mmHg. The patient undergoes a CT scan without transfer because the CT table can slide. The CT machine is an 80-slice multidetector device, PRIME Aquilion®, manufactured by Toshiba. The team leader can call a general surgeon, an anesthesiologist, a radiologist, an orthopedic surgeon, a plastic surgeon, or a neurosurgeon within 30 min any time during the day, if necessary. The leader decides whether to perform TBCT or selective CT on the trauma patient, not dependent on previous protocols presented [23–25]. This reduces the number of unnecessary scans for the patient receiving a head CT scan, preventing a disturbance of consciousness or head trauma.

Before the protocol was introduced, a patient received a conventional resuscitation room and trauma care based on the guidelines of the Japan Advanced Trauma Evaluation and Care program by the Advanced Trauma Life Support [2, 26]. Briefly, in the primary survey, the trauma care team begins with priority-oriented resuscitation. The team performs a focused assessment with sonography for trauma (FAST) with chest and pelvic X-ray examinations for diagnosis during the primary survey. In addition, if available in the facility, a selective CT scan is performed before emergency bleeding control is initiated. Each team leader decides whether or not to perform the CT scan if life-threating problems are clearly detected in the FAST and X-ray images or if patient transfer is difficult because of hemodynamic instability. The CT machine is located on the same floor as the resuscitation room, approximately 50 m away. The time required to perform the CT scan, including patient transfer time, is approximately 20 min.

This observational study utilized data from all trauma patients admitted to YCUMC. Our study included all trauma patients admitted to YCUMC between April 2014 and March 2016. Inclusion criteria consisted of all adult trauma patients (aged ≥ 18 years). Exclusion criteria included the following: patients with traumatic cardiopulmonary arrest on arrival, burn patients, patients who were < 18 years, and those who were transferred from other hospitals. We categorized the patients into two groups: patients treated using the conventional resuscitation room and patients treated in the CT-equipped trauma room.

The following data were retrospectively obtained from the patients’ medical records: sex; age; Abbreviated Injury Score (AIS); Injury Severity Score (ISS); Revised Trauma Score (RTS); probability of survival (Ps); initial vital signs upon arrival to the hospital, including HR, SBP, the Glasgow Coma Scale (GCS), respiratory rates, and body temperature; CT scans of the head and body trunk; initial laboratory data, including lactate, base excess, hemoglobin, fibrinogen, activated partial thromboplastin time, international normalized ratio of prothrombin time (PT-INR), fibrin degradation products, and D-dimer levels; injury mechanisms; intubation in ED; chest tube placement in ED; use of the resuscitative endovascular balloon for occlusion of the aorta in ED; transcatheter arterial embolization (TAE); need for large transfusions defined as transfused red blood cells of 10 units or more within 24 h after arrival to ED; ED stay, which was the time from arrival to transfer to the operation room, the angiography room, or the intensive care units (ICUs); time to CT scan, which was the time from arrival to the start of the CT scan; time to emergency operations to control bleeding, which was the time from arrival to the initiation of the operation; time to TAE, which was the time from arrival to the start of TAE; length of hospital stay (LOS; in days); and the length of ICU stay (in days). The types of trauma were categorized as blunt or penetrating. RTS was calculated using a formula described by Champion et al. [26, 27]. Ps was calculated using the Trauma and Injury Severity Score (TRISS) methods [28]. Hypotension was defined as SBP below 90 mmHg at arrival. Isolated traumatic brain injury was defined as having a GCS score of below 9 and an AIS head score of 3 or above without non-head region AIS score of greater than 1. Patients with multiple traumas were defined as those with an ISS of 16 or above.

The primary outcome measure was hospital mortality. Secondary outcome measures included LOS, length of ICU stay, need for large transfusions, time from the CT scan to the initiation of surgeries for controlling bleeding, time from the CT scan to the start of TAE, and the length of ED stay.

Data were analyzed for all eligible patients. Data were presented as median and interquartile ranges for not normally distributed values or number with percentages as appropriate. Continuous variables were compared between the two patient groups using the Mann–Whitney *U* test. Categorical variables were analyzed using Fisher’s exact test. Predictive survival rates (TRISS Ps), actual survival rates, and their ratios were calculated for the two groups: a patient group treated in the trauma room with CT and treated in the conventional resuscitation room. In order to compare predicted survival rate and actual survival rate by each group, Z statistic was calculated. M statistic was calculated to compare the difference from the standard severity distribution by Major Trauma Outcome Study (MTOS) [28]. As subgroup analysis, we calculated predictive survival rate, actual survival rate, Z statistic, and M statistic for multiple trauma patients, defined as ISS ≥ 16. In addition, we compared the two groups in terms of clinical and basic characteristics, such as mortality, age, and sex, to acknowledge the difference between the included and excluded samples.

Multivariate logistic regression analysis was used to control for potentially confounding variables, identified as prior to locating the CT in the trauma resuscitation room. Based on clinical reasoning and avoiding multicollinearity within variables, the following variables were entered in the model: CT machine in the trauma room, age, gender, ISS, RTS, lactate, PT-INR, and time to CT scan.

Furthermore, to minimize the effect of confounding variables due to a non-randomized study in evaluating the effect of locating a CT machine in the trauma resuscitation room on mortality, propensity scores were calculated with locating the CT machine or not as a dependent variable and ISS, RTS, sex, PT-INR, fibrinogen, and performing CT as independent variables. We used optimal methods to create 1:1-matched study groups with a 0.05 caliper width. After adjusting for these confounding variables, we performed both univariate and multivariate logistic regression analyses with a forward selection, in which *p* < 0.10 was set as a criterion to include in the model for evaluating the effect of locating the CT machine in the trauma resuscitation room on mortality.

A *p* value of < 0.05 was considered to indicate statistical significance. All statistical analyses were performed using EZR, which is a graphical user interface for R (version 3.1.2, The R Foundation for Statistical Computing, Vienna, Austria) [29] and IBM SPSS Statistics, Version 22.0 (IBM Corp, Armonk, NY, USA).

## Results

**Fig. 2:**
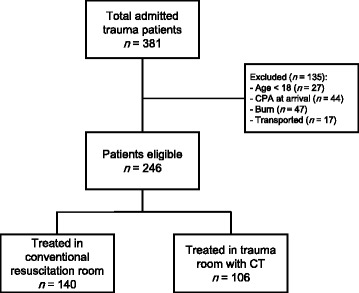
Study participant selection

During the study period, 381 trauma patients were admitted to YCUMC. We found a total of 246 trauma patients who were meeting the inclusion criteria (Fig. 2). We compared the included patient group with the excluded group. Compared to the included samples, the excluded samples had significantly higher mortality (34.8 vs. 5.7%; *p* < 0.001) and younger age [51 (36–69) years (median, IQR) vs. 45 (17–61); *p* < 0.001)]. We found no significant difference in sex (*p* = 0.482) between the groups.

Baseline characteristics of the included patients are summarized in Table 1. TRISS was applied to all 246 patients. In a total of 246 patients, the median age was 51 (36–69) years and the ISS was 10 (4–18). In total, 206 patients (83.7%) underwent CT and 15 died (6.1%). In the standard work-up group, one patient could not undergo a CT scan because of hemodynamic instability. The group with patients treated using the standard work-up included 140 patients, and the group with those treated in the trauma rooms equipped with a CT machine included 106 patients. There were no statistically significant differences in age, arrival status without GCS, type of trauma, isolated TBI using REBA, and urgent operations to control bleeding between the two groups. ISS (13.0 vs. 9.0; *p* = 0.002), CT scan of the head (78.3 vs. 66.4%; *p* = 0.046), CT scan of the body trunk (75.5 vs. 58.6%; *p* = 0.007), intubation in ED (48.1 vs. 30.7%; *p* = 0.08), and multiple trauma patients (47.2 vs. 30.0%; *p* = 0.08) were significantly higher in the group treated in the CT-equipped trauma room compared with the group treated using the standard work-up, respectively. There were no statistically significant differences in the hospital mortality. The median time to CT scan was significantly shorter after installation of the CT machine (23 vs. 37 min, *p* < 0.001). The median time in ED was significantly shorter in the group treated in the trauma room with a CT machine (72 vs. 91 min, *p* = 0.044). The median time to urgent operations to control bleeding and the time to TAE were not statistically different between the groups. LOS and the need for large transfusions were not significantly different between the two groups. TRISS Ps (96.75 vs. 97.80; *p* = 0.009) was significantly lower in the group treated in the CT-equipped trauma room (Table 1). The survival ratio in the main analysis was significantly higher in the CT trauma room group. The severity distribution was found far from the standard distribution of MTOS both in all patients and multiple trauma patients in the CT in the trauma room group (M statistic 0.78 and 0.39 respectively).

**Table 1:** Characteristics and outcome differences between patients treated in a trauma room with CT and a conventional resuscitation room (*n* = 246)

		Total (*n* = 246)	Conventional resuscitation room (*n* = 140)	Trauma room with CT (*n* = 106)	*p* value
		*n* (%)/median (IQR)	*n* (%)/median (IQR)	*n* (%)/median (IQR)	
Gender	Male	181 (73.6)	93 (66.4)	88 (83.0)	0.004
	Female	65 (26.4)	47 (33.6)	18 (17.0)	
Age		51 (36, 69)	50 (35, 70)	54 (38, 69)	0.769
Initial vital signs	GCS	14 (13, 15)	15 (14, 15)	14 (10, 15)	0.017
	Heart rate	88 (72, 103)	87 (72, 99)	89 (73, 105)	0.249
	Systolic pressure	141 (118, 162)	143 (121, 164)	139 (113, 159)	0.541
	Respiratory rate	20 (17, 24)	20 (17, 24)	20 (18, 24)	0.243
	Temperature	36.4 (36.1, 36.9)	36.4 (36.0, 36.9)	36.4 (36.1, 36.9)	0.618
CT performed		206 (83.7)	113 (80.7)	93 (87.7)	0.164
	For head	176 (71.5)	93 (66.4)	83 (78.3)	0.046
	For body trunk	162 (65.9)	82 (58.6)	80 (75.5)	0.007
Intubation in ER		94 (38.2)	43 (30.7)	51 (48.1)	0.008
Use REBOA		7 (2.8)	1 (0.7)	6 (5.7)	0.045
Arterial embolization		25 (10.2)	10 (7.1)	15 (14.2)	0.089
Place chest tube in ER		18 (7.5)	6 (4.5)	12 (11.3)	0.052
Type of Trauma	Blunt	209 (85.0)	118 (84.2)	91 (85.8)	0.857
	Penetrate	37 (15.0)	22 (15.7)	15 (14.1)	
Isolated TBI		21 (8.5)	12 (8.6)	9 (8.5)	1.000
Polytrauma		92 (37.4)	42 (30.0)	50 (47.2)	0.008
Hypotension		29 (11.8)	14 (10.0)	15 (14.2)	0.324
TAC INR > 1.3		16 (3.9)	5 (3.6)	11 (10.4)	0.038
ISS category	1–8	89 (36.2)	58 (41.4)	31 (29.2)	0.011
	9–15	65 (26.4)	40 (28.6)	25 (23.6)	
	16–24	54 (22.0)	29 (20.7)	25 (23.6)	
	≥ 25	38 (15.4)	13 (9.3)	25 (23.6)	
ISS		10 (4, 18)	9 (4, 16)	13 (5, 22)	0.002
RTS		7.84 (6.90, 7.84)	7.84 (7.48, 7.84)	7.84 (6.38, 7.84)	0.015
Ps		97.6 (92.2, 99.3)	97.8 (94.2, 99.4)	96.8 (81.3, 99.2)	0.009
Lactate		2.0 (1.3, 2.9)	2.0 (1.3, 2.9)	1.9 (1.3, 3.0)	0.876
BE		−0.20 (−2.62, 1.33)	−0.30 (−2.6, 1.2)	−0.10 (−2.8, 1.5)	0.807
Hg		13.2 (11.4, 14.4)	13.2 (11.6, 14.5)	13.2 (11.2, 14.0)	0.439
Fbg		286 (230, 336)	298 (257, 353)	260 (208, 314)	< 0.001
APTT		26.4 (24.1, 28.8)	26.4 (24.1, 28.5)	26.9 (24.3, 29.4)	0.283
PT-INR		1.04 (0.97, 1.12)	1.02 (0.95, 1.10)	1.06 (0.99, 1.15)	0.001
FDP		13.7 (3.6, 53.2)	10.8 (3.2, 30.9)	18.4 (4.7, 87.0)	0.025
D-dimer		7.6 (1.4, 29.4)	6.6 (1.2, 21.4)	9.7 (1.7, 46.1)	0.043
Transfusion	RBC	0 (0, 6)	0 (0, 4)	0 (0, 8)	0.009
	FFP	0 (0, 4)	0 (0, 0)	0 (0, 10)	0.002
Mortality	In-hospital	15 (6.1)	6 (4.3)	9 (8.5)	0.189
	24 h	6 (2.4)	2 (1.4)	4 (3.8)	0.407
RBC ≥ 10 U/24 h		39 (15.9)	17 (12.1)	22 (20.8)	0.079
Time to CT (min)		30 (23, 42)	37 (30, 48)	23 (18, 28)	< 0.001
Time to TAE (min)		81 (67, 93)	81 (56, 97)	80 (73, 87)	0.856
Time to operation (min)		94 (61, 122)	97 (7, 123)	83 (68, 121)	0.687
ED staying (min)		81 (58, 117)	91 (61, 122)	72 (53, 113)	0.044
ICU stay (day)		3 (2, 7)	3 (2, 7)	3 (2, 6)	0.811
Hospital stay (day)		16 (5, 35)	15 (5, 34)	17 (5, 37)	0.975

*TAC* traumatic acute coagulopathy, *ISS* Injury Severity Score, *RTS* Revised Trauma Score, *Ps* probability of survival

**Fig. 3:**
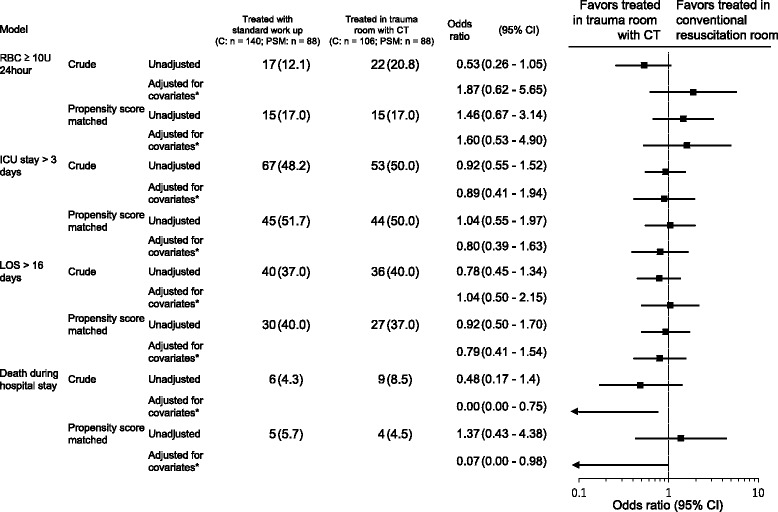
Results of multivariate logistic regression analysis and propensity score matched analysis on primary and secondary outcomes, comparing patients treated in the trauma room with CT and treated in the conventional resuscitation room. Adjusted models include age, gender, ISS, RTS, lactate, PT-INR, and time to CT as covariates

Multivariate logistic regression analysis was applied to control for potentially confounding variables. Being treated in the CT-equipped trauma room was an independent predictor for fewer hospital deaths (*p* = 0.04). Age, ISS, RTS, lactate, and time to CT scan were also independent predictors for hospital deaths (Table 2). Being treated in the trauma room with the CT machine was not a predictor for ICU stay (over 3 days), hospital stay (over 16 days), or the need for large transfusions. ISS was an independent predictor for ICU stay, hospital stay, and the need for large transfusions. RTS was an independent predictor for hospital stay and massive transfusions. Lactate was an independent predictor for massive transfusions. Details on the logistic regression results for ICU stay, hospital stay, and the need for large transfusions are provided (Fig. 3).

**Table 2:** Multivariate logistic regression analysis on mortality with associated factors (*n* = 246)

Factors		Odds ratio	(95% CI)	*p* value
Age		1.16	(1.02–1.33)	0.028
Gender	(reference: female)	1.65	(0.10–26.60)	0.720
ISS		1.20	(1.02–1.42)	0.029
RTS		0.11	(0.03–0.42)	0.002
Lactate		1.80	(1.04–3.11)	0.034
PT-INR		463	(0.51–42 × 10^4^)	0.077
Time to CT		0.84	(0.71–0.99)	0.037
Treated in trauma room with CT	(reference: treated in conventional resuscitation room)	0.002	(0.00–0.75)	0.040

After propensity score matching of patients treated using CT or not in the trauma room, we obtained 88 patients for each group with a total of 176 trauma patients. We found no significant differences in baseline characteristics, except for the time to perform CT and ED stay between the two matched study groups (Table 3). There were no statistically significant differences in hospital mortality on univariate analysis with the propensity score-matched samples. Furthermore, multivariate logistic regression analysis of the matched samples demonstrated that being treated in a CT-equipped trauma room was a significant factor and resulted in fewer hospital deaths [odds ratio (OR) 0.07, 95% confidence interval 0.00–0.98, *p* = 0.0478]. Age, ISS, and RTS were also significant independent predictors for hospital death (*p* < 0.001, *p* = 0.024, and *p* < 0.001, respectively; Table 4). Details of the results of logistic regression analysis after propensity score matching of ICU stay, hospital stay, and the need for large transfusions are shown in Fig. 3.

**Table 3:** Characteristics and outcome differences between patients treated in trauma room with CT and conventional resuscitation room after propensity score matching (*n* = 176)

		Total (*n* = 176)	Conventional resuscitation room (*n* = 88)	Trauma room with CT (*n* = 88)	*p* value
		*n* (%)/median (IQR)	*n* (%)/median (IQR)	*n* (%)/median (IQR)	
Gender	Male	145 (82.4)	72 (81.8)	73 (83.0)	1.000
	Female	31 (17.6)	16 (18.2)	15 (17.0)	
Age		50 (35.8,67.2)	49 (33, 68.3)	52.5 (36, 67)	0.726
Initial vital signs	GCS	14 (13, 15)	14 (13, 15)	14 (13.8, 15)	0.931
	Heart rate	88 (72, 103)	88 (72, 102.3)	89 (72.5, 103.1)	0.877
	Systolic pressure	142 (120, 162)	143 (117.8, 164.8)	140 (120, 159)	0.747
	Respiratory rate	20 (17, 24)	20 (17, 24)	20 (17, 24)	0.366
	Temperature	36.4 (36, 36.9)	36.4 (35.9, 36.8)	36.4 (36.1, 36.9)	0.537
CT performed		152 (86.4)	77 (87.5)	93 (85.2)	0.826
	For head	127 (72.2)	61 (69.3)	83 (75.0)	0.501
	For body trunk	122 (69.3)	58 (65.9)	80 (72.7)	0.414
Intubation in ER		72 (40.9)	34 (38.6)	38 (43.2)	0.646
Use REBOA		5 (2.8)	1 (1.1)	4 (4.5)	0.364
Arterial embolization		20 (11.4)	9 (10.2)	11 (12.5)	0.812
Place chest tube in ER		15 (8.5)	6 (6.8)	9 (10.2)	0.589
Type of trauma	Blunt	147 (83.5)	73 (83.0)	74 (84.1)	1.000
	Penetrate	29 (16.5)	15 (17.0)	14 (15.9)	
Isolated TBI		12 (6.8)	8 (9.1)	4 (4.5)	0.370
Polytrauma		69 (39.2)	35 (39.8)	34 (38.6)	1.000
Hypotension		20 (11.4)	10 (11.4)	10 (11.4)	1.000
TAC INR > 1.3		9 (5.1)	5 (5.7)	4 (4.5)	1.000
ISS category	1–8	62 (35.2)	32 (36.4)	30 (34.1)	0.696
	9–15	45 (25.6)	21 (23.9)	24 (27.3)	
	16–24	45 (22.2)	22 (25.0)	17 (19.3)	
	≥ 25	30 (17.0)	13 (14.8)	17 (19.3)	
ISS		10 (4.8, 20)	10 (4.8, 17.3)	10 (4.8, 20)	0.709
RTS		7.84 (6.90, 7.84)	7.84 (6.90, 7.84)	7.84 (7.06, 7.84)	0.873
Ps		97.7 (92.1, 99.3)	97.7 (92.1, 99.2)	97.6 (91.5, 99.3)	0.781
Lactate		2 (1.3, 2.9)	2 (1.4, 3.2)	1.9 (1.3, 2.7)	0.245
BE		−0.3 (−2.6, 1.5)	−0.5 (−2.9, 1.1)	0.15 (−2, 1.9)	0.082
Hg		13.3 (11.4, 14.6)	13.3 (11.2, 14.7)	13.3 (11.4, 14.3)	0.537
Fbg		280 (229, 327)	289 (246, 332)	268 (222, 317)	0.278
APTT		26.4 (24.1, 28.7)	26.2 (24.1, 28.6)	26.7 (24.1, 28.9)	0.771
PT-INR		1.04 (0.97, 1.12)	1.02 (0.95, 1.12)	1.06 (0.98, 1.12)	0.096
FDP		14 (3.9, 47.3)	15.3 (4.3, 37.8)	12.7 (3.9, 67.9)	0.858
D-dimer		7.6 (1.5, 25.8)	8.5 (1.5, 23.3)	7.1 (1.6, 35.6)	0.955
Transfusion	RBC	0 (0, 6)	0 (0, 6)	0 (0, 6)	0.909
	FFP	0 (0, 6)	0 (0, 4)	0 (0, 6)	0.727
Mortality	In-hospital	9 (5.1)	5 (5.7)	4 (4.5)	1.000
	24 h	3 (1.7)	2 (2.3)	1 (1.1)	1.000
RBC ≥ 10 U/24 h		30 (17.0)	15 (17.0)	15 (17.0)	1.000
Time to CT (min)		30 (22, 43)	40 (30, 52)	22 (17, 28)	< 0.001
Time to TAE (min)		81 (81, 100)	77 (56, 98)	81 (78, 91)	0.438
Time to operation (min)		75 (60, 122)	99 (60, 127)	83 (70, 118)	0.842
ED staying (min)		74 (56, 103)	91 (65, 115)	68 (52, 83)	< 0.001
ICU stay (day)		3 (2, 7)	3 (2, 9)	3 (2, 6)	0.401
Hospital stay (day)		17 (5, 39)	17 (5.5, 40)	17 (5, 37)	0.534

*TAC* traumatic acute coagulopathy, *ISS* Injury Severity Score, *RTS* Revised Trauma Score, *Ps* probability of survival

**Table 4:** Multivariate logistic regression analysis after propensity score matching on mortality with associated factors (*n* = 176)

Factors		Odds ratio	(95% CI)	*p* value
Age		1.14	(1.04–1.25)	< 0.001
ISS		1.16	(1.02–1.31)	0.024
RTS		0.09	(0.02–0.37)	< 0.001
Lactate		1.28	(0.979–1.67)	0.07
Treated in trauma room with CT	(reference: treated in conventional resuscitation room)	0.065	(0.00–0.985)	0.0487

## Discussion

Our study showed for the first time that a CT machine in the trauma room had a significantly positive effect on mortality. Patient mortality in the room with a CT machine was higher than that treated with the standard work-up; however, there were no statistically significant differences in hospital mortality after univariate analysis. This was after YCUMC was designated as a trauma center with more severe patients, higher ISS, and lower Ps. However, multivariate logistic regression analysis of the entire sample and the samples after propensity score matching showed positive effect on mortality. This significant association might attribute to a reduced time in decision-making. Equipping a trauma room with a CT machine allows clinicians quicker access to the machine to provide clinical decisions to treat faster with greater accuracy than a standard work-up. This speed with accuracy in the decision-making likely contributes to a lower mortality.

In addition, we had prepared and conducted series of simulation training in advance using the new trauma room with staffs involved in acute trauma care such as doctors, nurses, and laboratory technicians. Improvement of workflow through these simulation trainings might contribute to lower mortality for the trauma room with the CT group.

We did not find other significant associations in the secondary outcomes, such as the length of ICU stay and hospital stay. Our results suggest that the quality of care for patients will improve in a trauma room with a CT machine, independent of the severity of the trauma.

Previous studies with a pre–post study design showed inconsistent findings on the association between a CT machine in the trauma resuscitation room and an improvement in clinical outcomes [6, 17–19]. A randomized control trial, the RACT1 trial, compared a CT machine in the trauma room to the conventional resuscitation room in two Dutch trauma centers (*n* = 1124) and found no significant effects of the CT machine in the trauma room on patient mortality [21]. Recently, a multicenter randomized control trial to examine the effect of immediate TBCT scanning, the REACT 2 trial, conducted in several trauma centers found no significance in the reduction of mortality [30]; however, not all the trauma centers conducting the immediate TBCT had a CT machine in the trauma room. Stefan Huber-Wagner et al. compared the distance of the CT machine from the trauma resuscitation room with survival from several trauma centers [14]. Our study was the first to identify the significant and positive effect of a trauma room equipped with a CT machine on patient mortality.

Our study also showed that a CT machine in the trauma room reduced the time to the start of CT scan by 20 min (from 37 to 23 min) and the length of the ED stay by 19 min (from 91 to 72 min). In selected samples after propensity score matching, time to start CT scan was reduced by 18 min (from 40 to 22 min) and the length of the ED stay reduced by 25.5 min (from 94.5 to 69 min). This reduction was comparable or larger than that described in previous studies [7, 18, 20–22, 30]. This time saved could improve the workflow in a trauma care center and has a beneficial effect for the department staff. Previous studies have shown that the diagnostic work-up time was significantly longer in patients undergoing a conventional resuscitation [7, 18, 20–22, 30]. A rapid overview of all threatened body regions can be obtained, which increased decision-making and treatment times, leading to a lower mortality.

The presence of a CT machine in a trauma resuscitation room also has the following potential benefits. Installing a CT machine in a trauma room reduces the number of transfers to a CT room. Patient transfers can be time-consuming and laborious as the patient has to be moved to a transport stretcher and then back to a CT table. In a previous study, it was dangerous to transfer patients with hemodynamic instability to a CT room, which was called the tunnel of death [31]. These hemodynamically unstable patients could undergo a CT scan using the CT machine located in the trauma room. In our study, there were no patients who were unable to receive a CT scan because of hemodynamic instability in the group of patients treated in the trauma room with a CT machine. Installing a CT machine in a trauma room may resolve decision-making dilemmas in acute trauma care in patients without an obvious primary source or potentially multiple sources of hemorrhage. Such patients would benefit the most from CT scan information, as well as the reduced time to treatment.

We acknowledge several limitations of our study. First, this was a retrospective study; therefore, it was impossible to perform a sample size calculation. The current sample size would justify the utilization of a regression model with given proportions of outcomes [32]. Second, significant differences in baseline, including ISS and RTS, were observed between the two groups, which suggest heterogeneity in patients and raise concerns regarding the inability to control the effects of confounding factors. Thus, we employed a valid multivariate model to control these differences in our analysis. In addition, to overcome the bias, we further performed propensity score matching analysis because randomly allocating a patient into use or non-use of a trauma room with a CT machine could be difficult in certain clinical situations. Third, we also conducted a single-center study. There could be selection bias and a limitation in generalizability. Patients admitted to our hospital might be treated with a shorter time to transfer compared to those admitted in hospitals in a rural region. To evaluate the effects of a CT machine in the trauma room, our study design would be still appropriate. Fourth, we excluded pediatric patients and patients with CPA. This exclusion might affect the generalization of our study findings. Lastly, there was selection bias due to potential differences in decisions on performing the CT scan made by each trauma leader. The leaders were trained in YCUMC, and daily conferences by the trauma team could guarantee equality in decision-making.

## Conclusions

In conclusion, our study showed the effects on mortality using a CT-equipped trauma room. Our study also showed the time benefits of placing a CT machine in the trauma room. This time benefit could be critical in severe trauma patients, allowing life-threatening problems to be detected and allowing earlier critical decision-making. Installing a CT machine in the trauma room could reduce time for decision-making in treating a trauma patient and subsequently lower the mortality of trauma patients.

## Abbreviations

AIS: Abbreviated Injury Score
APTT: Activated partial thromboplastin time
CT: Computed tomography
ED: Emergency department
FAST: Focused assessment with sonography for trauma
Fbg: Fibrinogen
FDP: Fibrin degradation products
GCS: Glasgow Coma Scale
HR: Heart rate
ICUs: Intensive care units
ISS: Injury Severity Score
LOS: Length of hospital stay
MC council: Medical control council
MTOS: Major Trauma Outcome Study
Ps: Probability of survival
PT-INR: International normalized ratio of prothrombin time
RTS: Revised Trauma Score
SaO_2_: Percutaneous oxygen saturation
SBP: Systolic blood pressure
TAE: Transcatheter arterial embolization
TBCT: Total-body CT scanning
TRISS: Trauma and Injury Severity Score
YCUMC: The Yokohama City University Medical Center

## References

[1] World Health Organization. WHO. Injuries and violence: the facts 2014. http://www.who.int/violence_injury_prevention/en/. Accessed 18 Mar 2018.

[2] Committee of the Japan Association of Traumatology (2012). The Japan Advanced Trauma Evaluation and Care (JATEC).

[3] Salim A, Sangthong B, Martin M, Brown C, Plurad D, Demetriades D (2006). Whole body imaging in blunt multisystem trauma patients without obvious signs of injury: results of a prospective study. Arch Surg.

[4] Deunk J, Dekker HM, Brink M, van Vugt R, Edwards MJ, van Vugt AB (2007). The value of indicated computed tomography scan of the chest and abdomen in addition to the conventional radiologic work-up for blunt trauma patients. J Trauma.

[5] Pfeifer R, Pape HC (2008). Missed injuries in trauma patients: a literature review. Patient Saf Surg.

[6] Weninger P, Mauritz W, Fridrich P, Spitaler R, Figl M, Kern B (2007). Emergency room management of patients with blunt major trauma: evaluation of the multislice computed tomography protocol exemplified by an urban trauma center. J Trauma.

[7] Hilbert P, zur Nieden K, Hofmann GO, Hoeller I, Koch R, Stuttmann R (2007). New aspects in the emergency room management of critically injured patients: a multi-slice CT-oriented care algorithm. Injury.

[8] Bernhard M, Becker TK, Nowe T, Mohorovicic M, Sikinger M, Brenner T (2007). Introduction of a treatment algorithm can improve the early management of emergency patients in the resuscitation room. Resuscitation.

[9] Wurmb TE, Frühwald P, Hopfner W, Roewer N, Brederlau J (2007). Whole-body multislice computed tomography as the primary and sole diagnostic tool in patients with blunt trauma: searching for its appropriate indication. Am J Emerg Med.

[10] Yeguiayan JM, Yap A, Freysz M, Garrigue D, Jacquot C, Martin C (2012). Impact of whole-body computed tomography on mortality and surgical management of severe blunt trauma. Crit Care.

[11] Kanz KG, Paul AO, Lefering R, Kay MV, Kreimeier U, Linsenmaier U (2010). Trauma management incorporating focused assessment with computed tomography in trauma (FACTT) - potential effect on survival. J Trauma Manag Outcomes.

[12] Schoeneberg C, Schilling M, Burggraf M, Fochtmann U, Lendemans S (2014). Reduction in mortality in severely injured patients following the introduction of the “treatment of patients with severe and multiple injuries” guideline of the German society of trauma surgery—a retrospective analysis of a level 1 trauma center (2010-2012). Injury.

[13] Huber-Wagner S, Kanz KG, Mutschler W, Lefering R (2009). Primary pan-computed tomography for blunt multiple trauma: can the whole be better than its parts?. Injury.

[14] Huber-Wagner S, Lefering R, Qvick LM, Körner M, Kay MV, Pfeifer KJ (2009). Effect of whole-body CT during trauma resuscitation on survival: a retrospective, multicentre study. Lancet.

[15] Hutter M, Woltmann A, Hierholzer C, Gärtner C, Bühren V, Stengel D (2011). Association between a single-pass whole-body computed tomography policy and survival after blunt major trauma: a retrospective cohort study. Scand J Trauma Resusc Emerg Med.

[16] Wurmb TE, Frühwald P, Hopfner W, Keil T, Kredel M, Brederlau J (2009). Whole-body multislice computed tomography as the first line diagnostic tool in patients with multiple injuries: the focus on time. J Trauma.

[17] Wurmb TE, Quaisser C, Balling H, Kredel M, Muellenbach R, Kenn W (2011). Whole-body multislice computed tomography (MSCT) improves trauma care in patients requiring surgery after multiple trauma. Emerg Med J.

[18] Lee KL, Graham CA, Lam JM, Yeung JH, Ahuja AT, Rainer TH (2009). Impact on trauma patient management of installing a computed tomography scanner in the emergency department. Injury.

[19] Fung Kon Jin PH, Goslings JC, Ponsen KJ, van Kuijk C, Hoogerwerf N, Luitse JS (2008). Assessment of a new trauma workflow concept implementing a sliding CT scanner in the trauma room: the effect on workup times. J Trauma.

[20] Wada D, Nakamori Y, Yamakawa K, Fujimi S (2012). First clinical experience with IVR-CT system in the emergency room: positive impact on trauma workflow. Scand J Trauma Resusc Emerg Med..

[21] Saltzherr TP, Bakker FC, Beenen LF, Dijkgraaf MG, Reitsma JB, Goslings JC (2012). Randomized clinical trial comparing the effect of computed tomography in the trauma room versus the radiology department on injury outcomes (REACT-1). Br J Surg.

[22] Huber-Wagner S, Mand C, Ruchholtz S, Kühne CA, Holzapfel K, Kanz KG (2014). Effect of the localisation of the CT scanner during trauma resuscitation on survival—a retrospective, multicentre study. Injury.

[23] Mutze S, Madeja C, Paris S, Ostermann P, Ekkernkamp A (1999). Helical CT examination of multiple trauma patients in a digitized radiology department. Emerg Radiol.

[24] Hsiao KH, Dinh MM, McNamara KP, Bein KJ, Roncal S, Saade C (2013). Whole-body computed tomography in the initial assessment of trauma patients: is there optimal criteria for patient selection?. Emerg Med Australas.

[25] American College of Surgeons Committee on Trauma (2012). ATLS advanced trauma life support program for doctors. Student course manual.

[26] Champion HR, Sacco WJ, Carnazzo AJ, Copes W, Fouty WJ (1981). Trauma score. Crit Care Med.

[27] Champion HR, Sacco WJ, Copes WS, Gann DS, Gennarelli TA, Flanagan ME (1989). A revision of the trauma score. J Trauma.

[28] Boyd CR, Tolson MA, Copes WS (1987). Evaluating trauma care: the TRISS method. J Trauma.

[29] Kanda Y (2013). Investigation of the freely available easy-to-use software ‘EZR’ for medical statistics. Bone Marrow Transplant.

[30] Sierink JC, Treskes K, Edwards MJ, Beuker BJ, den Hartog D, Hohmann J (2016). Immediate total-body CT scanning versus conventional imaging and selective CT scanning in patients with severe trauma (REACT-2): a randomised controlled trial. Lancet.

[31] Mackay A (1999). Is the ‘tunnel of death’ a suitable modality for investigating the severely traumatized child?. ANZ J Surg.

[32] Hsieh FY, Block DA, Larsen MD (1998). A simple method of sample size calculation for linear and logistic regression. Stat Med.

